# An Unusual Splice Defect in the Mitofusin 2 Gene (*MFN2*) Is Associated with Degenerative Axonopathy in Tyrolean Grey Cattle

**DOI:** 10.1371/journal.pone.0018931

**Published:** 2011-04-15

**Authors:** Cord Drögemüller, Ursula Reichart, Torsten Seuberlich, Anna Oevermann, Martin Baumgartner, Kathrin Kühni Boghenbor, Michael H. Stoffel, Claudia Syring, Mireille Meylan, Simone Müller, Mathias Müller, Birgit Gredler, Johann Sölkner, Tosso Leeb

**Affiliations:** 1 Institute of Genetics, University of Bern, Bern, Switzerland; 2 Institute of Animal Breeding and Genetics, University of Veterinary Medicine Vienna, Vienna, Austria; 3 Neurocenter, Division of Experimental Clinical Research, University of Bern, Bern, Switzerland; 4 Division of Molecular Pathobiology, University of Bern, Bern, Switzerland; 5 Division of Veterinary Anatomy, University of Bern, Bern, Switzerland; 6 Clinic for Ruminants, University of Bern, Bern, Switzerland; 7 Division of Livestock Sciences, University of Natural Resources and Applied Life Sciences, Vienna, Austria; Instituto de Ciencia de Materiales de Madrid - Instituto de Biomedicina de Valencia, Spain

## Abstract

Tyrolean Grey cattle represent a local breed with a population size of ∼5000 registered cows. In 2003, a previously unknown neurological disorder was recognized in Tyrolean Grey cattle. The clinical signs of the disorder are similar to those of bovine progressive degenerative myeloencephalopathy (weaver syndrome) in Brown Swiss cattle but occur much earlier in life. The neuropathological investigation of an affected calf showed axonal degeneration in the central nervous system (CNS) and femoral nerve. The pedigrees of the affected calves suggested a monogenic autosomal recessive inheritance. We localized the responsible mutation to a 1.9 Mb interval on chromosome 16 by genome-wide association and haplotype mapping. The *MFN2* gene located in this interval encodes mitofusin 2, a mitochondrial membrane protein. A heritable human axonal neuropathy, Charcot-Marie-Tooth disease-2A2 (CMT2A2), is caused by *MFN2* mutations. Therefore, we considered *MFN2* a positional and functional candidate gene and performed mutation analysis in affected and control Tyrolean Grey cattle. We did not find any non-synonymous variants. However, we identified a perfectly associated silent SNP in the coding region of exon 20 of the *MFN2* gene. This SNP is located within a putative exonic splice enhancer (ESE) and the variant allele leads to partial retention of the entire intron 19 and a premature stop codon in the aberrant *MFN2* transcript. Thus we have identified a highly unusual splicing defect, where an exonic single base exchange leads to the retention of the preceding intron. This splicing defect represents a potential explanation for the observed degenerative axonopathy. Marker assisted selection can now be used to eliminate degenerative axonopathy from Tyrolean Grey cattle.

## Introduction

Spontaneous mutants in domestic animals provide insights on genotype-phenotype correlations, which are relevant for biomedical research [1]. Disorders of the nervous system may be caused by trauma, infectious agents, toxins, metabolic aberrations, and also genetic defects. Many inherited disorders of the nervous system are known in domestic animals including cattle [2]. Strong inbreeding in the bovine population has increased the risk of the occurrence of genetic diseases. The most common mode of transmission of genetic defects in cattle is monogenic autosomal recessive inheritance [2], [3]. Calves with recessive defects are typically the consequence of inbreeding. This is common in livestock breeding programs when female descendants of artificial insemination sires are mated with test bulls that are also direct descendents of the former sires. Recessive genetic diseases become apparent many years after the initial mutation event. In cattle, most of the known recessive defects became apparent 5 to 10 generations after the founder animal, which corresponds to the time after which male and female descendants of the original carrier are mated. During the latent phase the deleterious allele might have been widely spread throughout the population explaining sudden outbreaks with many affected animals appearing simultaneously [3].

Currently, there are six known examples of inherited neurological/neuromuscular diseases of cattle where the molecular basis has been elucidated and gene tests for selection against the deleterious mutation are available. Three of these defects segregate in the international Brown Swiss cattle population. The bovine progressive degenerative myeloencephalopathy (weaver syndrome) is characterized by axonal degeneration and vacuolation of the white matter of the spinal cord and degenerative changes or numeric reduction of the Purkinje cells in the cerebellum. Affected animals become clinically apparent at about 6–8 months of age. The *weaver* mutation was mapped to cattle chromosome (BTA) 13 in the year 1993, but until now the causative mutation has not been identified [4]. Spinal muscular atrophy (SMA) is characterized by severe muscular atrophy, progressive quadriparesis, and sternal recumbency due to degeneration of ventral motor neurons in the spinal cord. First symptoms of SMA appear at 3–4 weeks of age and this progressive lethal disease is caused by a nonsense mutation of *FVT1* [5]. Spinal dysmyelination (SDM) is characterized by pathological reduction of myelin in specific tracts of the spinal cord and severe neurological symptoms where the causative mutation in *SPAST* has recently been identified [6]. Affected calves usually die or are euthanized during the first week of life. In Belgian Blue cattle the causative mutations for congenital muscular dystony type 2 (CMD2) has been identified in the *SLC6A5* gene encoding an ion channel for the neurotransmitter glycine [3]. A clinically related phenotype termed CMD1 in Belgian Blue cattle or pseudomyotonia in Chianina cattle is caused by two different mutations in the *ATPA2A1* gene encoding a calcium pump in the sarcoplasmic reticulum of skeletal muscle [3], [7], [8]. Other cattle breeds also show inherited diseases affecting the nervous systems but the molecular basis is unclear [2].

Tyrolean Grey cattle represent a local breed with a population size of ∼5000 registered cows. In 2003, a previously unknown neurological disorder was recognized in Tyrolean Grey cattle by the veterinary practitioner Dr. Florian Demetz. The clinical signs of the so-called Demetz syndrome are similar to those of weaver syndrome in Brown Swiss cattle but occur earlier in life. The pedigrees of the affected calves suggest a monogenic autosomal recessive mode of inheritance and nearly all affected animals trace back to a single female. The neuropathology of this disease is characterized by an axonal degeneration targeting primarily the spinal cord. This study reports the positional cloning and subsequent identification of a candidate causative mutation.

## Results

### Clinical and neuropathological findings

**Figure 1:**
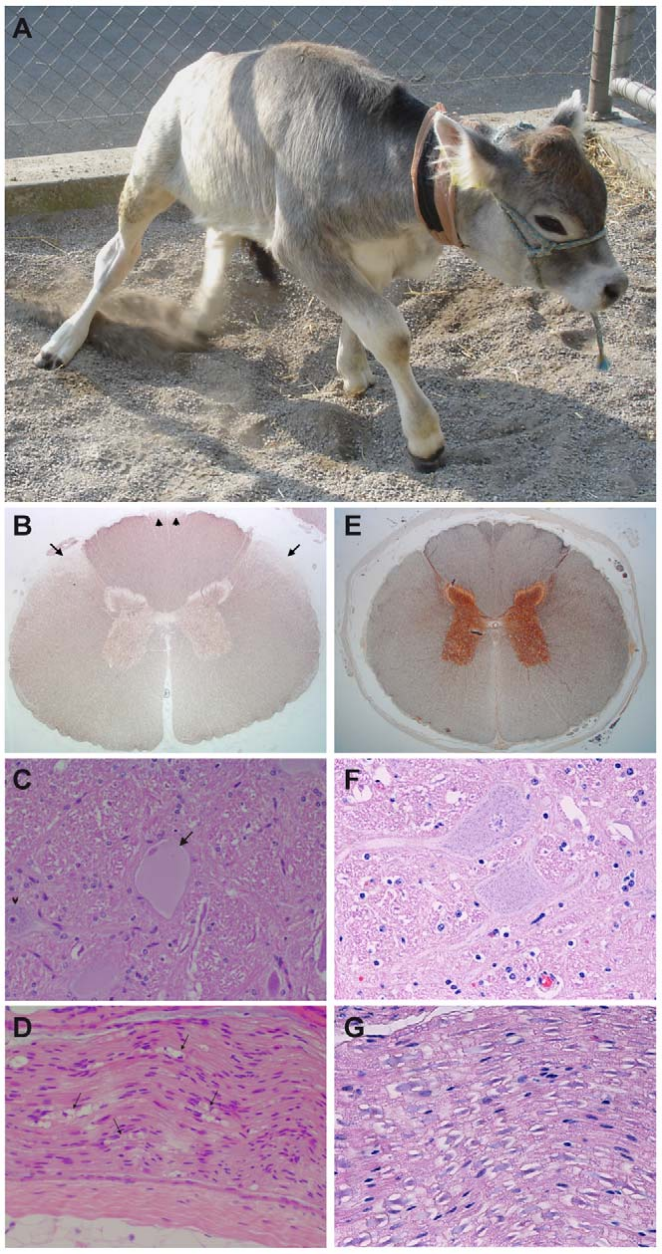
Phenotype of degenerative axonopathy in Tyrolean Grey cattle. (**A**) Moderate to severe spinal ataxia in an affected calf. Gait abnormalities and proprioceptive deficits were present in all limbs, but were markedly more pronounced in the hind than in the front limbs. (**B**) Thoracic spinal cord of a calf affected with degenerative axonopathy. Bilateral-symmetrical axonal loss leading to reduction of axonal density in the dorsal spinocerebellar tract (arrows) and gracile fascicle (arrowheads). Modified Bielschowsky staining, 12.5× magnification. (**C**) Swollen and chromatolytic neuron (arrow) adjacent to a normal neuron (arrowhead) in the mesencephalic red nucleus. The nucleus is displaced at the periphery and pyknotic. H&E, 400× magnification. (**D**) Femoral nerve. Wallerian type axonal degeneration and axonal loss: digestion chambers containing axonal and myelin fragments (arrows), proliferation of Schwann cells forming bands of Büngner. H&E, 400× magnification. (**E**, **F**, **G**) Corresponding tissue sections of a normal age-matched control animal.

We obtained one affected calf for clinical and neuropathological examination, which is described in more detail elsewhere [9]. Spinal ataxia was the most prominent feature (Figure 1). The animal showed mild ambulatory paraparesis with moderate to severe ataxia most pronounced in the pelvic limbs. All four extremities were affected, but gait abnormalities and conscious proprioceptive deficits were markedly more severe in the pelvic than in the thoracic limbs. The calf often stood with a wide stance in all limbs, otherwise it lost balance after a few seconds and fell to the ground (Video S1). According to the Tyrolean Grey cattle breeding organizations affected calves show progressive ataxia in the hind legs, starting at the age of 1 to 1.5 months. Progression of the symptoms eventually leads to recumbency. Most affected animals have to be slaughtered between 8 and 10 months of age.

The neuropathological examination of this affected calf revealed prominent bilateral-symmetrical (Wallerian type) axonal degeneration/loss and astrogliosis targeting primarily the dorsal spinocerebellar tract and the gracile fascicle of the spinal cord. Scattered degenerating axons were observed in the remaining white matter tracts of the spinal cord, in the white matter of the brain and in the femoral nerve. Additionally, swelling and chromatolysis of most neurons in the red nucleus and occasional motor neurons in brainstem nuclei and spinal cord ventral horns was observed. Lesions were compatible with a multisystem axonal degeneration (“long tract degeneration”) and their pattern together with neuronal changes suggest a distal axonopathy due to primary neuronal metabolic dysfunction. We found a similar degenerative axonopathy in archived material from three other Tyrolean Grey calves.

### Analysis of the mode of inheritance

We identified 49 cases in Tyrolean Grey cattle and obtained DNA samples from 44 of them. The pedigree records were consistent with a monogenic autosomal recessive inheritance (Figure S1). According to the pedigree records the mutation event occurred before the year 1972.

### Mapping of the causative mutation

**Figure 2:**
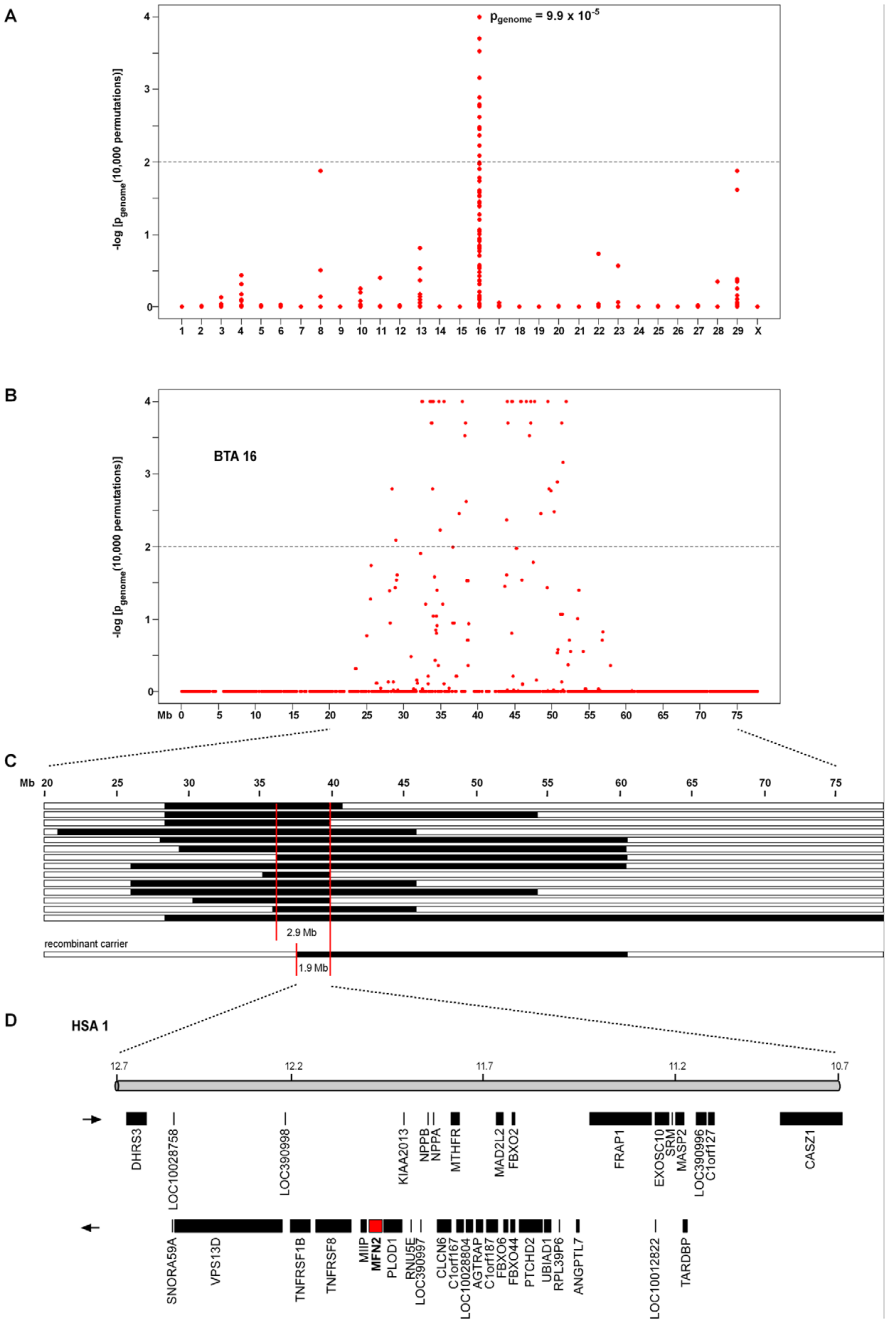
Genome-wide association and homozygosity mapping of Tyrolean Grey cattle degenerative axonopathy. (**A**) Case-control genome-wide association analysis shows a significant association to SNPs on chromosome 16. (**B**) Single SNP association results across BTA 16. (**C**) Homozygosity mapping of the degenerative axonopathy mutation. The analysis of SNP genotypes from 14 affected calves indicated an extended shared homozygous region of 2.9 Mb. One recombinant chromosome from a carrier animal further narrowed the critical interval to 1.9 Mb. (**D**) Gene content of the corresponding human chromosome 1 segment.

We genotyped 54,001 evenly spaced SNPs on 14 affected calves and 27 controls. After removing 19,506 non-informative markers 34,495 SNPs were used for the subsequent analyses. In a genome-wide association study (GWAS) several SNPs on BTA16 were found to be associated with degenerative axonopathy (Figure 2A). The associated SNPs were spread over a 25 Mb region. The best-associated SNP had a genome-wide corrected p-value of 9.9×10^−5^ (Figure 2B). No other region in the genome showed genome-wide associated SNPs.

Subsequently, we applied a homozygosity mapping approach to fine-map the region containing the degenerative axonopathy mutation. Based on the pedigree records we hypothesized that the affected calves most likely were inbred to one single founder animal (Figure S1). Under this scenario the affected individuals were expected to be identical by descent (IBD) for the causative mutation and flanking chromosomal segments. We analyzed the cases for extended regions of homozygosity with simultaneous allele sharing. Only one genome region fulfilled our search criteria. On BTA 16 all 14 affected genotyped calves were homozygous and shared identical alleles over 46 SNP markers corresponding to a 2.88 Mb interval from 36.87–39.75 Mb (Figure 2C).

In order to further narrow down the critical interval, we then examined 18 obligate carriers for the mutation and reconstructed one copy of the disease-associated haplotype in each animal. One of the carriers showed a recombination event, which allowed us to narrow down the critical interval harboring the causative mutation to 1.90 Mb from 37.85–39.75 Mb (Figure 2C).

### Identification and characterization of functional candidate genes

As the annotation of the bovine genome is still far from perfect, we inferred the gene content of the mapped interval from the corresponding human interval (Figure 2D). The mapped degenerative axonopathy interval corresponds to a segment from 10.7–12.7 Mb on HSA 1. This human interval contains 34 annotated genes and 4 hypothetical loci (NCBI MapViewer, build 37.1). A careful inspection of these genes and database searches of their presumed function revealed the genes encoding TAR DNA binding protein (*TARDBP*), methylenetetrahydrofolate reductase (*MTHFR*), and mitofusin 2 *(MFN2*) as possible functional candidate genes within the critical interval. Mutations in any of these three genes can cause neurological disease in humans [10].

### Genomic mutation analysis

Initially, we sequenced all coding exons of the *TARDBP, MTHFR*, and *MFN2* genes in three affected and five control animals. We did not find any polymorphism in the six exons of the *TARDBP* gene and its expression was similar in tissue samples from a case and a control animal (Figure S2). In the 12 exons and adjacent intron regions of the *MTHFR* gene, we identified 11 polymorphisms but none of them was perfectly associated with the disease in the eight animals of our mutation analysis. In the *MFN2* gene, we identified four SNPs in the coding sequence (Table S1). As all of these SNPs were translationally silent, we re-sequenced a contiguous genomic interval of more than 37 kb spanning the complete *MFN2* gene including 8 kb of 5′-flanking sequence and parts of the preceding gene. In this 37 kb interval we identified 174 polymorphisms between the eight Tyrolean Grey cattle and the bovine reference genome sequence (Table S1). Only one of these polymorphisms, the synonymous SNP c.2229C>T located in exon 20 of the *MFN2* gene, was perfectly associated with the phenotype in the eight animals. We then genotyped 485 Tyrolean Grey cattle for this SNP and found again an almost perfect association of the mutant T-allele with the degenerative axonopathy phenotype (Table 1). All cases were homozygous for the mutant allele and none of the controls were homozygous for this allele. Only one reported carrier yielded a discordant genotyping result and was homozygous for the wildtype allele, which is most likely due to a phenotyping error of the affected offspring (Table 1). The mutant allele was absent from 83 healthy cattle from 10 genetically diverse breeds. Southern blots from an affected and a control calf yielded the expected bands and did not indicate any copy number changes or structural variations of the *MFN2* gene (data not shown).

**Table 1:** Association of the *MFN2* mutation with the degenerative axonopathy phenotype.

		Tyrolean Grey cattle			Other breeds
*MFN2*		affected(n = 44)	carrier^a^(n = 45)	controls(n = 396)	controls(n = 83)
**c.2229C>T**	**CC**		**1** b	**358**	**83**
	**CT**		**44**	**38**	
	**TT**	**44**			

a Cattle recorded as parent of affected calves.

b For this animal only one single suspicious calf was recorded by the breeding organization and the diagnosis of this calf had not been confirmed by necropsy. Genotyping of additional flanking markers revealed that this animal did not carry the disease-associated haplotype. Therefore, the reported calf most likely represented a phenocopy and the parent is indeed free of the deleterious mutation.

### Transcript analysis

As the associated *MFN2* c.2229C>T polymorphism did not change the encoded amino acid sequence, we analyzed the *MFN2* transcripts in four different tissues from an affected calf and a control animal, which was homozygous wildtype at the c.2229C>T SNP. Northern blotting revealed the expected 4.6 kb band in both animals. RT-PCR experiments showed that similar to human two alternatively spliced *MFN2* transcripts are expressed in cattle. The two transcript isoforms differ by the presence or absence of the untranslated 85 bp exon 3, which is not resolved in the Northern blot. The two alternatively spliced transcripts encode identical proteins. Both transcript isoforms were detected in the affected and control calf.

**Figure 3:**
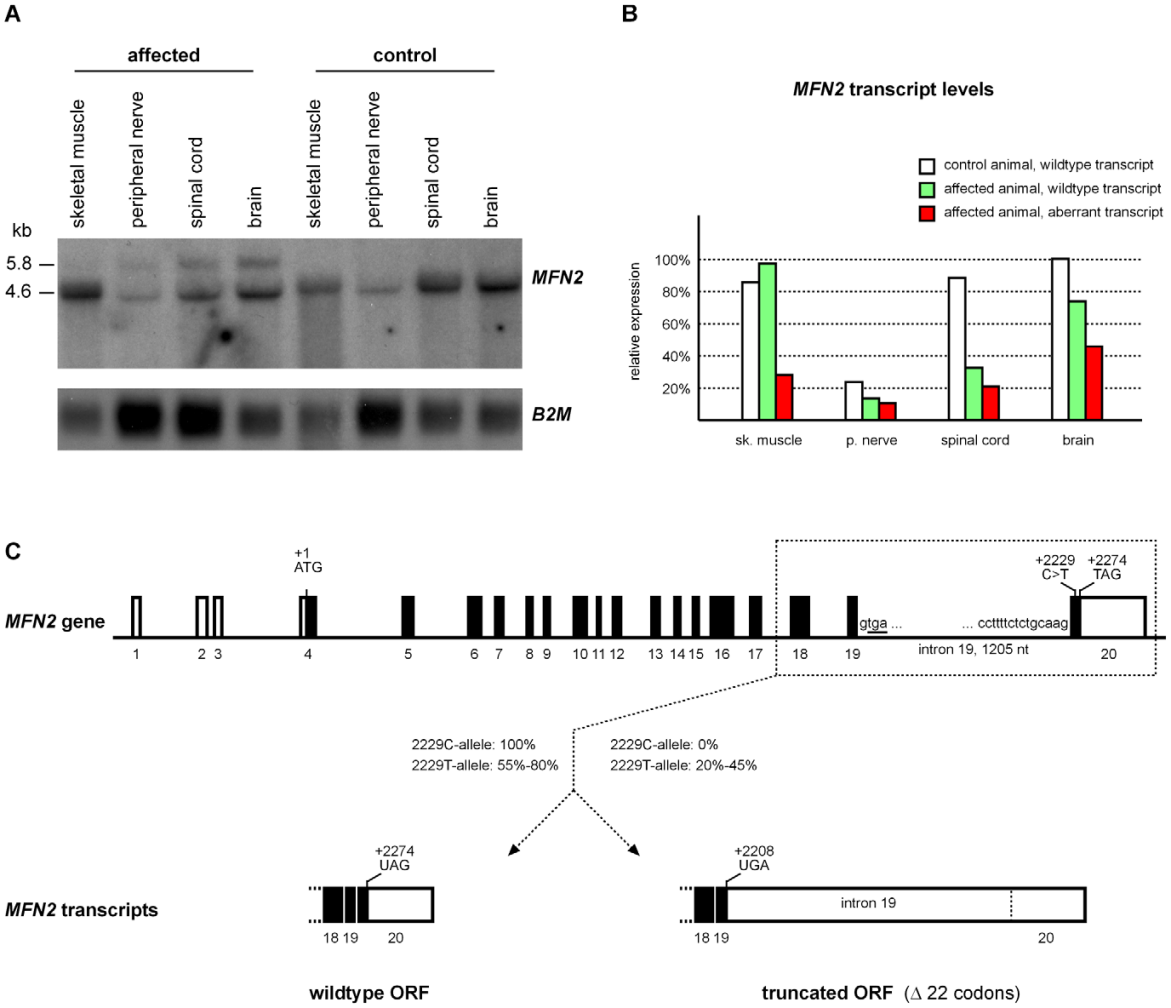
*MFN2* transcript analysis. (**A**) Northern blot using a full length *MFN2* cDNA probe. Note the additional band of 5.8 kb in all examined tissues of an affected calf. (**B**) Relative *MFN2* transcript levels. The *MFN2* expression levels were normalized to *BM2* expression. For the affected calf the relative expression levels of the wildtype and mutant transcript are indicated. (**C**) Bovine *MFN2* gene organization. The 2229C-wildtype allele gives rise to a transcript, which is spliced as expected. The mutant 2229T-allele leads to partial retention of intron 19. Thus from the mutant allele tissue-specific varying fractions of wildtype transcript and an aberrant transcript encoding a truncated MFN2 protein are expressed.

In the affected calf, there was, however, also an additional larger transcript of 5.8 kb present in all investigated tissues (Figure 3A). The mRNA expression level of *MFN2* varied across the investigated tissues with the highest expression seen in brain and about four-fold lower expression in peripheral nerves (Figure 3B). The total amount of *MFN2* transcript was comparable between the affected and the control calf. However, in the affected calf, depending on the tissue between 20% and 45% of the *MFN2* transcripts corresponded to the 5.8 kb transcript. We performed RT-PCR on the RNA samples from both animals and sequenced the products to confirm their identity. The additional 5.8 kb band in the Northern blot corresponds to an *MFN2* transcript, in which the 1,205 nt intron 19 is retained (Figure 3C). As there is a stop codon immediately at the beginning of intron 19, the aberrant 5.8 kb transcript encodes a C-terminally truncated protein, which is predicted to lack 22 amino acids compared to the wildtype MFN2 protein (p.N736X; Figure S3). Transcript analysis in a heterozygous carrier animal revealed that the intron retention occurs only in cis on transcripts with the mutant T-allele, but not in trans on the wildtype transcripts (Figure S4).

### Protein Analysis

**Figure 4:**
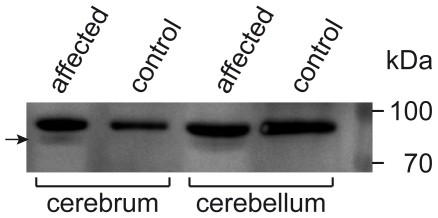
Western immunoblot analysis of MFN2. Extracts from the cerebral and cerebellar cortex of a degenerative axonopathy affected calf and a healthy control calf were analyzed. In the samples from the affected calf a weak additional MFN2 band of slightly lower molecular mass (83.7 kDa, arrow) as compared to the full-length wild-type protein (86.3 kDa) is visible. Conversely, in the healthy control only the wild-type MFN2 is detected. Molecular masses of the size standard are indicated on the right.

The molecular masses of wild-type bovine MFN2 and its putative C- terminally truncated variant were calculated at 86.3 kDa and 83.7 kDa, respectively. To investigate whether the aberrant transcript is translated, we analyzed nervous tissues of the affected calf and the control calf by Western immunoblot using a polyclonal anti-MFN2 rabbit antiserum. While in the control calf only the full-length wild-type MFN2 was identified, the affected calf showed full-length MFN2 as well as a faint band of slightly lower molecular mass, which was in the range of that expected for the truncated MFN2 (Figure 4).

### Electron microscopy of mitochondria

**Figure 5:**
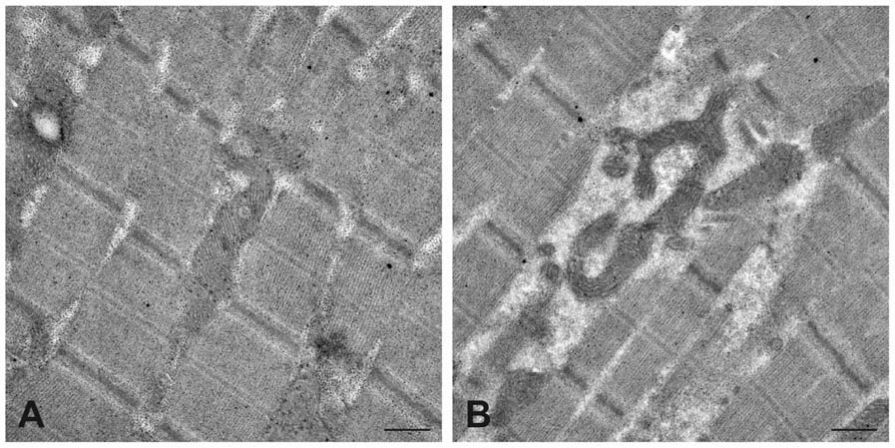
Electron micrographs of striated muscle fibers. (**A**) Muscle fibers of healthy calf contain rigorously oriented, elongate mitochondria. (**B**) Uneven space between myofibrils in striated muscle of affected calf displays branched mitochondria. (scale bar  = 0.5 µm)

Mitochondria were well-delineated by an outer membrane and the inner membrane displayed typical cristae. Though basically tubular in shape, branching of mitochondria appeared to be more prominent in an affected calf as compared to a control animal (Figure 5). This observation was particularly noticeable in striated muscle fibers.

## Discussion

In this study we performed a comprehensive genetic investigation of inherited degenerative axonopathy in Tyrolean Grey cattle. With the availability of genome sequences and high-density SNP genotyping microarrays the mapping of causative mutations for monogenic traits in domestic animal species has become a fairly straightforward procedure [3], [11]. Thus, we could quickly identify a relatively small interval for the degenerative axonopathy mutation in Tyrolean Grey cattle containing less than 40 genes. The *MFN2* gene stood out as a good functional and positional candidate gene. Based on the recessive mode of inheritance, we expected to find a loss-of-function mutation affecting the coding sequence of *MFN2*. Therefore, we initially did not rate the synonymous c.2229C>T SNP as promising candidate causative mutation. Only when we systematically screened the complete *MFN2* gene including the complete 5′-flanking sequence up to the next flanking gene, we realized that c.2229C>T was the only *MFN2* variant that showed the expected association to the degenerative axonopathy phenotype. This mutation is located at the 25^th^ nucleotide of the last exon, which did not immediately strike us as potentially important region for splicing. However, the comprehensive analysis of genomic DNA and mRNA by different experimental approaches including the traditional Northern blot clearly demonstrated that the c.2229C>T variant does indeed lead to a partial retention of the entire intron 19. During the last years it has become increasingly clear that splice site recognition in mammals is not only dependent on the conserved 5′- and 3′-splice site sequences of the introns. It rather is also dependent on exon recognition and additional splicing factors. The c.2229C>T mutation is located in a potential exonic splice enhancer (ESE) motif [12]. The T-allele eliminates a potential binding site for the splicing factor ASF/SF-2 and it potentially increases the affinity for SC35, a different auxiliary splicing protein (Figure S5).

The c.2229C>T variant affects a CpG dinucleotide, which are known to have a relatively high mutation rate in mammals. Interestingly, the same C>T transition must have independently occurred more than once during evolution as several species including humans have the T-allele at this position, which is found in cattle with degenerative axonopathy. Apparently, in these other species the splicing of *MFN2* transcripts functions normally. This further highlights the complexity of exon recognition in higher eukaryotes.

In human patients with hereditary diseases, other examples of translationally silent exonic mutations that affect the splicing were reported before [13], [14]. As these mutations cannot be as easily recognized as non-synonymous mutations there is always the risk that they remain unidentified. This becomes especially important in next generation sequencing projects, where typically thousands of genomic variants are initially identified. Currently, many researchers consider only the non-synonymous variants in such projects as promising candidates [15]. Thus other types of mutations such as translationally silent ESE mutations may easily be missed. Therefore, bioinformatic tools that can identify potentially deleterious mutations with an effect on splicing will be very important to make optimal use of the new large-scale re-sequencing approaches.

The c.2229C>T mutation affects the splicing of the *MFN2* transcripts. Our findings indicate that in homozygous mutant animals, about 20% to 45% of the *MFN2* transcripts retain the last intron. These aberrant transcripts contain an open reading frame lacking the last 22 codons (Figure S3). The wildtype MFN2 is a 757 amino acid transmembrane protein anchored in the outer mitochondrial membrane [16], [17]. The vast majority of the protein including N-terminus and C-terminus are located in the cytoplasm and only two amino acids between the transmembrane domains are located inside the mitochondria. The protein can form homo-and heterodimers via the interaction of its two heptad repeat regions that can make up coiled-coil structures [18].

Mitochondria are very dynamic organelles that continuously undergo fusions and fissions. Dimerization mediated by MFN2 is an essential step in the tethering and subsequent fusion of mitochondria [19]. Additionally, MFN2 is present in the endoplasmic reticulum (ER) and required for tethering of mitochondrial and ER membranes [20]. About 60 protein-changing MFN2 mutations have been described in human patients with Charcot-Marie-Tooth disease 2A2 [21]–[25], which is characterized by axonal degeneration that affects peripheral neurons with the longest axons. However, in contrast to degenerative axonopathy in Tyrolean Grey cattle, no axonal degeneration of the central nervous system has been described in humans. The pathophysiology of this degenerative axonopathy in cattle remains unknown. In mouse models, Mfn2 mutations cause massive axonal degeneration in motor nerves and impair mitochondrial fusion leading to uneven distribution of mitochondria within neuronal processes [26], [27]. However, the mechanisms how mitochondrial fusion affects neuronal function and axonal survival remain unclear [28]. It has been hypothesized that defects in mitochondrial fusion result in irregular distribution of mitochondrial DNA between organelles leading to loss of mitochondrial DNA and impairment of oxidative phosphorylation [21]. Damaged mitochondria are abnormally shaped and incorrectly transported leading to mitochondrial accumulations within axons. Accordingly, in human patients with Charcot-Marie-Tooth disease 2A2 due to MFN2 mutations, axonal degeneration is associated with mitochondrial changes as shown by electron microscopy. These include focal mitochondrial accumulations within distal axons and degeneration with mitochondrial swelling, condensation and irregularities of mitochondrial membranes [24], [25], [29]. Although we did not assess ultrastructural changes morphometrically in the present study, the qualitative evidence of enhanced branching patterns in the affected animal is compatible with the contention that the reported mutation had an effect on mitochondrial shape.

The cattle MFN2 mutation truncates the last 22 amino acids. We do not know whether this mutant protein is further modified *in vivo*. One of the human Charcot-Marie-Tooth mutations, p.G751X, is predicted to truncate the last seven amino acids of the protein and confirms that these residues are essential for normal MFN2 function [25]. All the reported human MFN2 mutations act in an autosomal dominant mode of inheritance, whereas the bovine mutation is inherited recessively. We think that this is due to the fact that from the mutant bovine allele only a relatively small fraction of aberrant protein is expressed. Thus, assuming equal translation efficiencies of the different transcripts, even homozygous mutant Tyrolean Grey cattle produce more than 50% of the normal amount of wildtype protein and only 20%–45% of the mutant protein. This may explain that the degenerative axonopathy manifests only in homozygous mutant animals. Additionally, cattle are not as intensively monitored as humans. Therefore, it is quite possible that very mild neurological symptoms in heterozygous mutant animals have not been recognized in the past.

Our genotyping results of 396 healthy Tyrolean Grey cattle indicate that the current carrier frequency in this population is close to 10%. This number may be somewhat inflated as breeders submit preferentially descendants of known carriers to genetic testing. However, pedigree analysis reveals that 7.2% of the genome in Tyrolean Grey cattle born 2004–2008 is derived from the important ancestor that has transmitted the deleterious allele to most of the cases. This animal was traced in 90.2% of all pedigrees. Our findings emphasize how the breeding practice in modern livestock populations can lead to high frequencies of deleterious alleles within a few generations. Therefore, it is essential that domestic animal populations with small effective population sizes are continuously monitored for the appearance of recessive defects, so that selection against the deleterious alleles can be implemented as early as possible.

In conclusion, we have identified a translationally silent variant in a potential exonic splice enhancer of the bovine *MFN2* gene as the causative mutation for a splice defect. The knowledge on *MFN2* mutations in human Charcot-Marie-Tooth patents suggests that this splice defect is also responsible for the inherited degenerative axonopathy in Tyrolean Grey cattle, although we have no functional proof for this claim. Our finding enables genetic testing and the eradication of this genetic disease from the breeding population.

## Materials and Methods

### Animals

The animal experiments in this study were carried out under permit 58/09 (canton of Bern) and in strict accordance with all regulations for the work with animals at the University of Bern. We collected samples from 44 degenerative axonopathy affected calves (22 male, 27 female) from different farms. The phenotype classification was primarily based on the owners' reports. Some affected animals were seen by a veterinarian and for five cases necropsy results and a histopathological examination were available. Additionally, we collected 45 samples recorded as parents (21 sires, 24 dams) of affected offspring and 396 healthy Tyrolean Grey cattle resulting in a total of 485 samples from this breed. For the association study we also used 83 healthy cattle from 10 genetically diverse *Bos taurus* breeds.

### Neuropathological examination

Brain, spinal cord and tissue samples from the peripheral nerves and skeletal muscle were immersion fixed in 4% neutral buffered formaldehyde. Representative tissue samples were processed, embedded in paraffin, sectioned at 5 µm and stained with hematoxylin and eosin (HE). Selected sections of the brain and spinal cord were stained with Luxol fast blue HE and modified Bielschowsky stain.

### DNA and RNA extraction

Genomic DNA was isolated from semen or tissue using the Nucleon Bacc2 kit (GE Healthcare). Total RNA was isolated using Trizol reagent according to the manufacturer's instructions (Invitrogen).

### Mapping of the degenerative axonopathy mutation

Genomic DNA from 14 cases, 27 controls, and 18 carriers was genotyped using Illumina's BovineSNP50 BeadChip with 54,001 SNPs [30], [31]. The results were analyzed with PLINK [32]. After removing 1,588 SNPs with low genotyping success (failed calls > 0.1) the average genotyping rate per individual was 99.9%. A total of 18,239 SNPs had a minor allele frequency (MAF) <0.1 and were removed from the dataset. A case-control analysis using the options –assoc was applied. Genome-wide corrected empirical p-values were determined applying the max(T) permutation procedure implemented in PLINK with 10,000 permutations. To identify extended homozygous regions with allele sharing across all affected animals the options –homozyg-group and –homozyg-match were applied. Tracing of the disease-associated haplotype in parents of non-genotpyed cases was performed with Fortran software developed for that purpose. Homozygosity of carriers for the alternative allele was used to fine-map the critical interval as described before [33]. All given positions correspond to the Btau4.0 cattle genome assembly. The corresponding human chromosome segment (build 37.1) was identified by BLASTN searches of bovine SNP flanking sequences to the human genome sequence.

### Genomic DNA analysis

Genomic DNA from 3 cases, 2 carriers, and 3 unrelated healthy calves, which were assumed to be homozygous free of the mutation were used for mutation analysis. PCR products were amplified using AmpliTaqGold360Mastermix (Applied Biosystems). PCR products were directly sequenced on an ABI 3730 capillary sequencer (Applied Biosystems) after treatment with exonuclease I and shrimp alkaline phosphatase. Sequence data were analyzed with Sequencher 4.9 (GeneCodes). The bovine reference genome sequence contained a gap in the region of the *MFN2* gene. We determined a complete and contiguous sequence of the bovine *MFN2* gene as well as of two alternatively spliced transcripts and deposited these sequences in the EMBL database (accessions FN868562–FN868564). Potential exonic splice enhancer (ESE) motifs were detected with ESE finder 3.0 [34].

### Northern Blot

For the Northern blot 20 µg total RNA of various tissues derived from an affected as well a healthy control animal were denatured by Glyoxal/DMSO and separated by gel electrophoresis. RNA was transferred to Hybond™-N+ membrane (GE Healthcare) by capillary blotting. Hybridization was carried out as previously described [35] using either a ^32^P-labeled full length *MFN2* cDNA probe or a probe containing nt 24-670 of the bovine beta-2-microglobulin cDNA (*B2M*) (accession NM_173893) as a loading control. The density of the individual bands was measured on an AlphaImager™ gel documentation system (Cell Biosciences).

### RT-PCR

We performed cDNA synthesis using SuperScriptIII (Invitrogen) and an oligo d(T)24V primer. For the RT-PCR we used SequalPrep Long Polymerase (Invitrogen), a forward primer located in exon 2 (5′-GCGGAGTCATTCAGTAGCCATCTT-3′), and a reverse primer located in the 3′-UTR of exon 20 (5′-AGTTGGAAGGTCTTCCTGCAACAG-3′) to amplify the 2521/2606/3726/3811 bp products containing the entire *MFN2* open reading frame. These products correspond to wildtype isoforms 1/2, and mutant isoforms1/2 including intron 19, respectively. To confirm the identity of the alternatively spliced transcripts in the affected animal we sequenced the obtained PCR products with internal primers located in exon 19, intron 19 and exon 20, respectively. Furthermore we performed RT-PCR using a forward primer located in exon 15 (5′-TGATGGGCTACAGTGACCAG-3′) and a reverse primer located in intron 19 (5′-ATGGTCTCAGCAGGAGATGG-3′) to confirm the retention of intron 19 in the affected calf.

### Western immunoblot

Nervous tissue samples of the affected calf and the control calf were homogenized at a ratio of 1∶10 (w/v) in 5% (v/v) glucose solution using a Fastprep blender (Bio-Rad). Proteins were extracted from the homogenates by boiling in equal amounts of Lämmli Buffer (Bio-Rad), separated by SDS-PAGE in an 8% hand-casted acrylamide gel (7 cm × 6 cm) and transferred to a polyvinylidene fluoride membrane. Next, MFN2 was detected by using a polyclonal rabbit-anti-MFN2 (N-terminal) serum (Sigma), a goat anti-rabbit IgG-HRP conjugate (DAKO) as secondary antibody and a chemiluminescence-based detection system (ECL plus, GE healthcare). A molecular mass standard (See-Blue, Invitrogen) was included in one lane of the gel.

### Electron microscopy

Samples from striated muscles, peripheral nerves, kidney, salivary glands, brain and spinal cord were collected immediately after euthanasia. Tissue cubes of 1-2 mm edge length were immersion fixed at room temperature for 2 h. The fixative was made up of 2.5% glutaraldehyde in 0.1 M cacodylate buffer with 2 mM CaCl_2_, pH 7.4. After two washing steps in the same buffer, samples were postfixed with 1% (w/v) OsO_4_ in cacodylate buffer for 2 h at 4°C, washed again, dehydrated and embedded in an Epon-Araldite mixture according to standard protocols. Ultrathin sections of 70 nm in thickness were double stained with 0.5% uranyl acetate and 0.5% lead citrate and examined in a Zeiss transmission EM 109 at 50 kV.

## Supporting Information

Figure S1 Pedigrees of families in study. Filled symbols represent degenerative axonopathy affected calves, open symbols represent normal cattle. DNA samples were available only for the numbered individuals. Animals genotyped on the SNP chip are indicated by asterisks. Parents of affected offspring and animals genotyped as carriers of the *MFN2* c.2229C>T mutation are shown with half-filled symbols. The mutation was predominantly distributed by three offspring (A, B, C) of a single cow. Most of the mothers of affected calves are related to these three offspring and the number of generations is indicated. A recent pedigree (D) indicates that the causative mutation might be older.
(PDF)

Figure S2 Northern blot using a full length *TARDBP* cDNA probe. The expression of *TARDBP* is similar between tissues from an affected and a control calf. Normalization was performed by hybridization with a *B2M* probe.
(PDF)

Figure S3 Multispecies alignment of MFN2 protein sequences. Sequences correspond to Genbank accessions from self-predicted (cattle), NP_055689 (human), XP_544564 (dog), NP_573464 (mouse), NP_570964 (rat), NP_001084869 (Xenopus), NP_001121726 (zebrafish). Note the extremely high sequence conservation from human to zebrafish. Important functional motifs as annotated in the UniProtKB/Swiss-Prot accession O95140 are highlighted in color. The x-ray structure of a partial human MFN1 protein suggests that the C-termini of mitofusins utilize a heptad repeat region (HR2) to form dimeric antiparallel coiled coils. The HR2 region of MFN2 contains amino acids 693–754. The aberrant MFN2 transcript observed in affected Tyrolean Grey cattle encodes a protein lacking the last 22 amino acids (boxed). Thus the aberrant MFN2 protein lacks a large part of the functionally important HR2 domain.
(PDF)

Figure S4 Sequence analysis of *MFN2* transcripts from a heterozygous carrier animal. (**A**) We amplified a cDNA fragment containing the retained intron from a carrier animal by using a forward primer located in intron 19 and a reverse primer located in exon 20 on oligo-dT primed cDNA. The sequence analysis of this RT-PCR product shows only the mutant T-allele at the c.2229 position. This indicates that the splicing aberration occurs only in transcripts from the mutant allele and not in transcripts from the wildtype allele. (**B**) A sequencing reaction using the same primers on a genomic PCR product from the same carrier animal confirms the presence of both alleles on the genomic DNA.
(PDF)

Figure S5 Sequence context of the c.2229C>T polymorphism. (**A**) The last 30 nucleotides of intron 19 (lowercase letters) and the first 70 nucleotides including the stop codon of exon 20 (uppercase letters) are shown. The candidate causative mutation is located at the 25th base of exon 20. (**B**) Analysis of exonic splice enhancer elements (ESEs). The c.2229C>T mutation eliminates a potential binding site for the SF2/ASF splicing factor and increases the binding score for the splicing factor SC35. The binding scores for the different splicing factors for the first 40 nucleotides of exon 20 were calculated with the program ESE Finder 3.0. Binding scores for different splicing proteins are based on different weight matrices and cannot be compared directly to each other.
(PDF)

Table S1 Polymorphisms and genotypes of 8 Tyrolean Grey cattle in the region of the *MFN2* gene.
(XLSX)

Video S1 Ataxia in a Tyrolean Grey calf affected by degenerative axonopathy.
(WMV)
