# Supplementary figures and images for: An Unusual Splice Defect in the Mitofusin 2 Gene (MFN2) Is Associated with Degenerative Axonopathy in Tyrolean Grey Cattle
Source: PLoS One. 2011 Apr 15;6(4):e18931. doi: 10.1371/journal.pone.0018931 (PMC3078137; doi:10.1371/journal.pone.0018931)

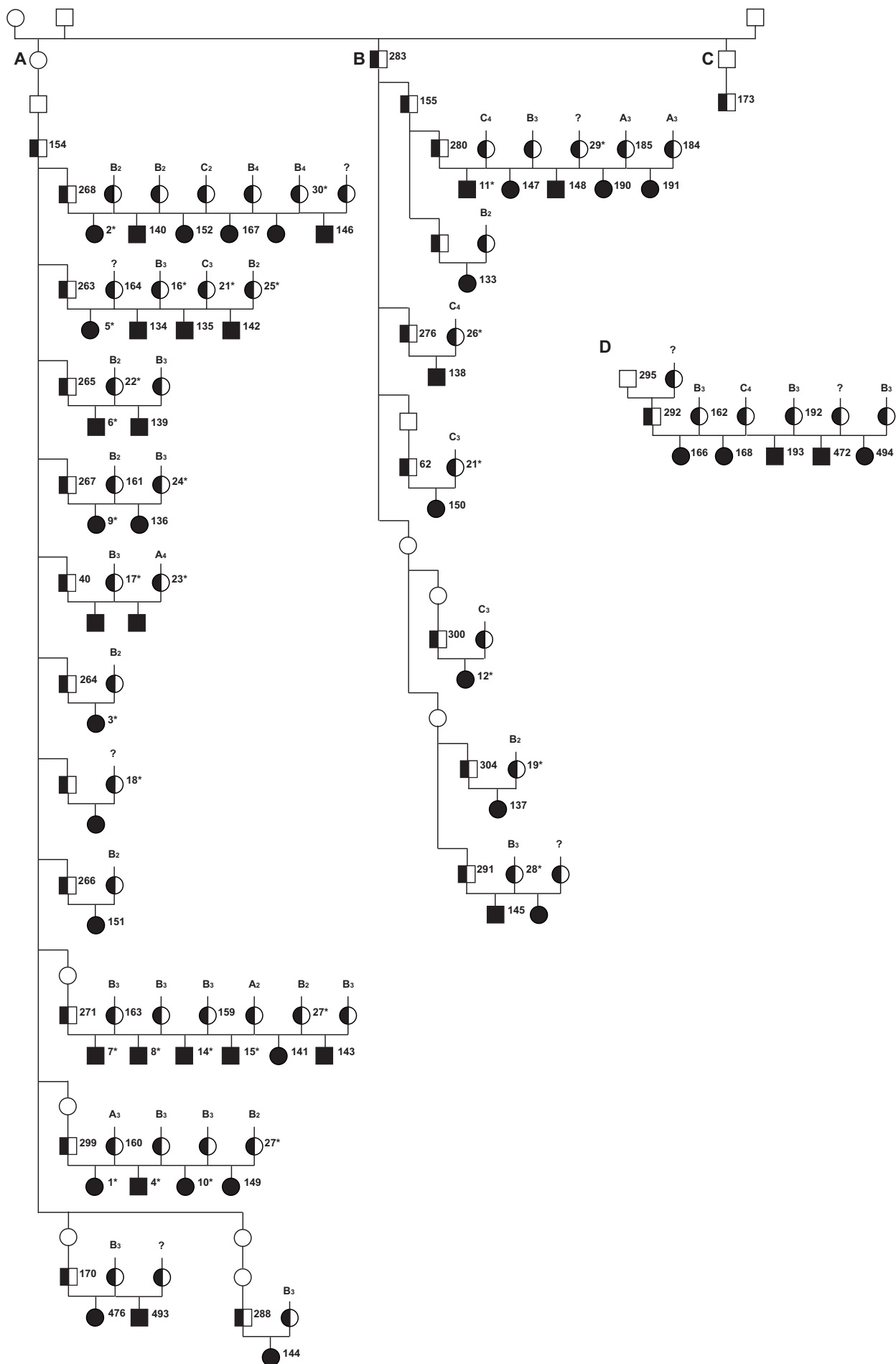

Supplement: Figure S1 — Pedigrees of families in study. Filled symbols represent degenerative axonopathy affected calves, open symbols represent normal cattle. DNA samples were available only for the numbered individuals. Animals genotyped on the SNP chip are indicated by asterisks. Parents of affected offspring and animals genotyped as carriers of the MFN2 c.2229C>T mutation are shown with half-filled symbols. The mutation was predominantly distributed by three offspring (A, B, C) of a single cow. Most of the mothers of affected calves are related to these three offspring and the number of generations is indicated. A recent pedigree (D) indicates that the causative mutation might be older. (PDF) [file pone.0018931.s001.pdf]

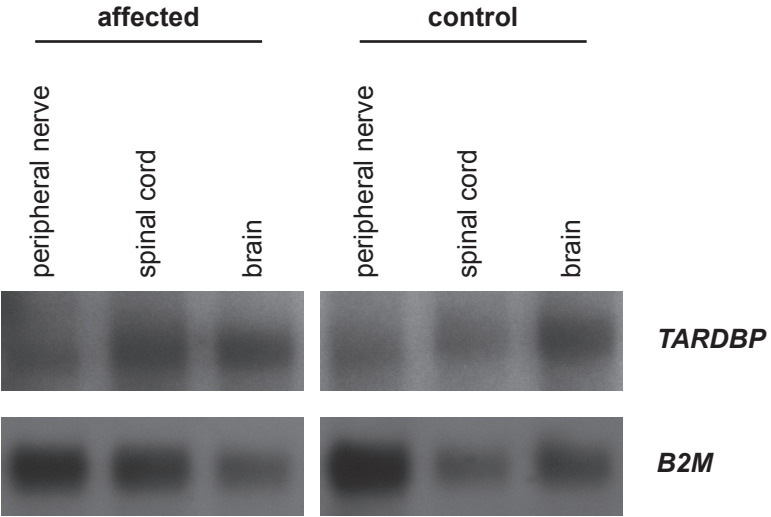

Supplement: Figure S2 — Northern blot using a full length TARDBP cDNA probe. The expression of TARDBP is similar between tissues from an affected and a control calf. Normalization was performed by hybridization with a B2M probe. (PDF) [file pone.0018931.s002.pdf]

**A** cDNA

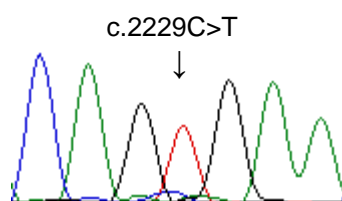

**B** genomic DNA

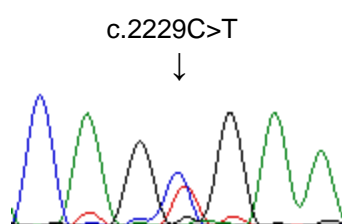

Supplement: Figure S4 — Sequence analysis of MFN2 transcripts from a heterozygous carrier animal. (A) We amplified a cDNA fragment containing the retained intron from a carrier animal by using a forward primer located in intron 19 and a reverse primer located in exon 20 on oligo-dT primed cDNA. The sequence analysis of this RT-PCR product shows only the mutant T-allele at the c.2229 position. This indicates that the splicing aberration occurs only in transcripts from the mutant allele and not in transcripts from the wildtype allele. (B) A sequencing reaction using the same primers on a genomic PCR product from the same carrier animal confirms the presence of both alleles on the genomic DNA. (PDF) [file pone.0018931.s004.pdf]
