# Supplementary material for: An Unusual Splice Defect in the Mitofusin 2 Gene (MFN2) Is Associated with Degenerative Axonopathy in Tyrolean Grey Cattle
Source: PLoS One. 2011 Apr 15;6(4):e18931. doi: 10.1371/journal.pone.0018931 (PMC3078137; doi:10.1371/journal.pone.0018931)
Supplement: Figure S5 — Sequence context of the c.2229C>T polymorphism. (A) The last 30 nucleotides of intron 19 (lowercase letters) and the first 70 nucleotides including the stop codon of exon 20 (uppercase letters) are shown. The candidate causative mutation is located at the 25th base of exon 20. (B) Analysis of exonic splice enhancer elements (ESEs). The c.2229C>T mutation eliminates a potential binding site for the SF2/ASF splicing factor and increases the binding score for the splicing factor SC35. The binding scores for the different splicing factors for the first 40 nucleotides of exon 20 were calculated with the program ESE Finder 3.0. Binding scores for different splicing proteins are based on different weight matrices and cannot be compared directly to each other. (PDF) [file pone.0018931.s005.pdf]

A

c.2229C>T

tgccactaca aggtaacctt ttctctgcag GAATAAAGCC GGCTGGTTGG ACAGCGAACT CAACATGTTC ACCCACCAGT ACCTGCAGCC CAGCAGATAG

B

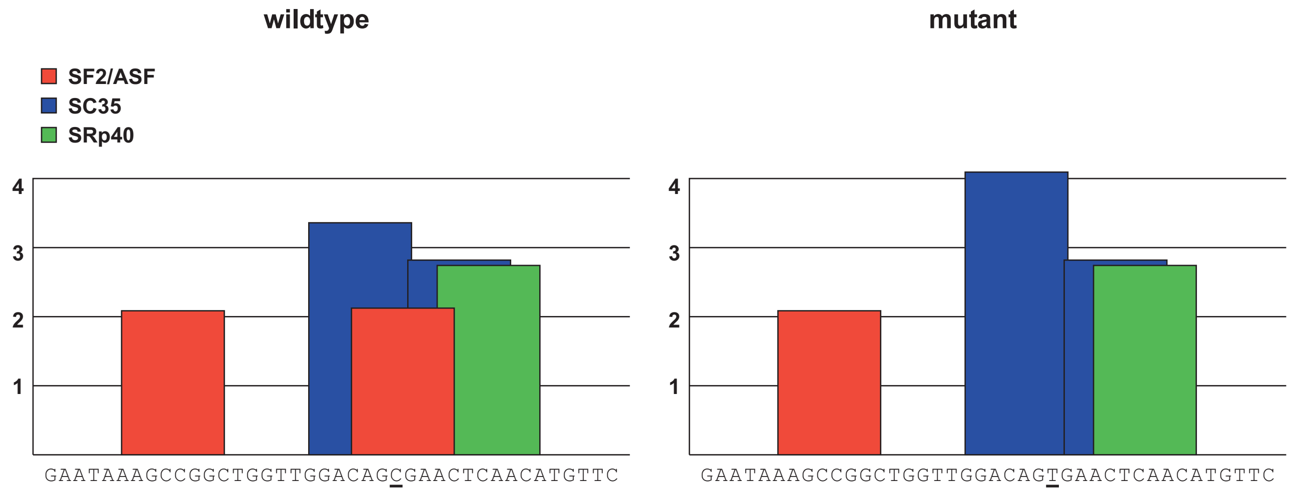

Splicing factor	Position	Binding site	Score (wildtype/mutant)
SF2/ASF	6-12	AAGCCGG	2.12 / 2.12
SF2/ASF	22-28	CAGCGAA	2.17 / --
SC35	19-26	GGACAGCG	3.41 / 4.05
SC35	26-33	GAACTCAA	2.85 / 2.85
SRp40	28-34	ACTCAAC	2.82 / 2.82
