# Supplementary material for: An Unusual Splice Defect in the Mitofusin 2 Gene (MFN2) Is Associated with Degenerative Axonopathy in Tyrolean Grey Cattle
Source: PLoS One. 2011 Apr 15;6(4):e18931. doi: 10.1371/journal.pone.0018931 (PMC3078137; doi:10.1371/journal.pone.0018931)
Supplement: Figure S3 — Multispecies alignment of MFN2 protein sequences. Sequences correspond to Genbank accessions from self-predicted (cattle), NP_055689 (human), XP_544564 (dog), NP_573464 (mouse), NP_570964 (rat), NP_001084869 (Xenopus), NP_001121726 (zebrafish). Note the extremely high sequence conservation from human to zebrafish. Important functional motifs as annotated in the UniProtKB/Swiss-Prot accession O95140 are highlighted in color. The x-ray structure of a partial human MFN1 protein suggests that the C-termini of mitofusins utilize a heptad repeat region (HR2) to form dimeric antiparallel coiled coils. The HR2 region of MFN2 contains amino acids 693–754. The aberrant MFN2 transcript observed in affected Tyrolean Grey cattle encodes a protein lacking the last 22 amino acids (boxed). Thus the aberrant MFN2 protein lacks a large part of the functionally important HR2 domain. (PDF) [file pone.0018931.s003.pdf]

```
Cattle     MSLLFSRCNSIVTVKKDKRHMAEVNASPLKHFVTAKKKINGIFEQLGAYIQESASFLEDTHRNTELDPVTTEEQVLDVKGYLSKVRGISEVLARRHMKVA 100
Human      MSLLFSRCNSIVTVKKDKRHMAEVNASPLKHFVTAKKKINGIFEQLGAYIQESAGFLEDTHRNTELDPVTTEEQVLDVKGYLSKVRGISEVLARRHMKVA 100
Dog        MSLLFSRCNSIVTVKKNKRHMAEVNASPLKHFVTAKKKINGIFEQLGAYIQESATFLEDTYRNAELDPVTTEEQVLDVKGYLSKVRGISEVLARRHMKVA 100
Mouse      MSLLFPRCNSIVTVKKDKRHMAEVNASPLKHFVTAKKKINGIFEQLGAYIQESATFLEDTYRNAELDPVTTEEQVLDVKGYLSKVRGISEVLARRHMKVA 100
Rat        MSLLFSRCNSIVTVKKDKRHMAEVNASPLKHFVTAKKKINGIFEQLGAYIQESATFLEETYRNAELDPVTTEEQVLDVKGYLSKVRGISEVLARRHMKVA 100
Xenopus    MSVLFSRPAIKVTTKKDKRFMAEVTASPLKHFVTAKKKINGIFDQLGAYTQESYNFLEETYKNAELDPVASEEQVLEVKCYLTNVAGISEVLARRHMKVA 100
Zebrafish  MSLVFPRPNTSVIGKKDKRLMAEVNASPLKHFVTAKKKINGIFEQLGAYIKESSAFLEETYSNEELDPVTTEEQVAEVRGYLSKVAGIGEVLSRRHMKVV 100
           **::*.*     *  **:** ****.******************:***** :**  ***:*: * *****::**** :*: **::* **.***:******.

  tripartite GTP-binding motif of the GTPase domain
Cattle     FFGRTSNGKSTVINAMLWDKVLPSGIGHTTNCFLRVGGTDGHEAFLLTEGSEEKKSVKTVNQLAHALHQDEQLHAGSMVSVMWPNSKCPLLKDDLVLMDS 200
Human      FFGRTSNGKSTVINAMLWDKVLPSGIGHTTNCFLRVGGTDGHEAFLLTEGSEEKKSVKTVNQLAHALHQDEQLHAGSLVSVMWPNSKCPLLKDDLVLMDS 200
Dog        FFGRTSNGKSTVINAMLWDKVLPSGIGHTTNCFLRVEGTDGHEAFLLTEGSEEKRSAKTVNQLAHALHQDKQLHAGSLVSVMWPNSKCPLLKDDLVLMDS 200
Mouse      FFGRTSNGKSTVINAMLWDKVLPSGIGHTTNCFLRVEGTDGHEAFLLTEGSEEKRSVKTVNQLAHALHQDEQLHAGSLVSVMWPNSKCPLLKDDLVLMDS 200
Rat        FFGRTSNGKSTVINAMLWDKVLLSGIGHTTNCFLRVEGTDGHEAFLLTEGSEEKRSVKTVNQLAHALHQDEQLHAGSLVSVMWPNSKCPLLKDDLVLMDS 200
Xenopus    FFGRTSNGKSSVINAMLWDKVLPSGIGHTTNCFLRVEGTDGNDAYLLTDGSEEKRSVKTVNQLAHALHQDDLLDSGCMVSVMWPNSKCPLLKDDLVLMDS 200
Zebrafish  FFGRTSNGKSSVINAMLWDKVLPSGIGHTTNCFLRVEGTDGNESFLLTEGSDERKSVKTVNQLAHALHQDEDLDAGSLVCVMWPKAKCALLRDDLVLVDS 200
           **********:*********** ************* ****::::***:**:*::*.*************. *.:*.:*.****::**.**:*****:**

Cattle     PGIDVTTELDSWIDKFCLDADVFVLVANSESTLMQTEKQFFHKVSERLSRPNIFILNNRWDASASEPEYMEEVRRQHMERCTSFLVDELGVVDRAQAGDR 300
Human      PGIDVTTELDSWIDKFCLDADVFVLVANSESTLMQTEKQFFHKVSERLSRPNIFILNNRWDASASEPEYMEEVRRQHMERCTSFLVDELGVVDRAQAGDR 300
Dog        PGIDVTTELDSWIDKFCLDADVFVLVANSESTLMQTEKHFFHKVSERLSRPNIFILNNRWDASASEPEYMEEVRRQHMERCTSFLVDELGVVDRSQAGDR 300
Mouse      PGIDVTTELDSWIDKFCLDADVFVLVANSESTLMQTEKQFFHKVSERLSRPNIFILNNRWDASASEPEYMEEVRRQHMERCTSFLVDELGVVDRGQAGDR 300
Rat        PGIDVTTELDSWIDKFCLDADVFVLVANSESTLMQTEKQFFHKVSERLSRPNIFILNNRWDASASEPEYMEEVRRQHMERCTSFLVDELGVVDRGQAGDR 300
Xenopus    PGIDVTTELDSWIDKFCLDADVFVLVANSESTLMQTEKQFFHKVNERLSRPNIFILNNRWDASASEPEYMEEVRRQHMERCTSFLVDELGVLDRTQAGDR 300
Zebrafish  PGIDVTTELDSWIDKFCLDADVFVLVANSESTLMQTEKSFFHKVNERLSSPNIFILNNRWDASANEPEYMEEVRRQHMDRCTSFLVDELRVVDRSHAGDR 300
           ************************************** *****.**** *************.*************:********** *:** :****

Cattle     IFFVSAKEVLSARVQKAQGMPEGGGALAEGFQVRMFEFQNFERQFEECISQSAVKTKFEQHTVRAKQIAEAVRLIMDSLHIAAQEQRVYCLEMREERQDR 400
Human      IFFVSAKEVLSARVQKAQGMPEGGGALAEGFQVRMFEFQNFERRFEECISQSAVKTKFEQHTVRAKQIAEAVRLIMDSLHIAAQEQRVYCLEMREERQDR 400
Dog        IFFVSAKEVLNARIQKAQGMPEGGGALAEGFQVRMFEFQNFERRFEECISQSAVKTKFEQHTVRAKQIAEAVRLIMDSLHMAAREQQVYCEEMREERQDR 400
Mouse      IFFVSAKEVLNARIQKAQGMPEGGGALAEGFQVRMFEFQNFERRFEECISQSAVKTKFEQHTVRAKQIAEAVRLIMDSLHIAAQEQRVYCLEMREERQER 400
Rat        IFFVSAKEVLNARIQKAQGMPEGGGALAEGFQVRMFEFQNFERRFEECISQSAVKTKFEQHTVRAKQIAEAVRLIMDSLHIAAQEQRVYCLETREERQER 400
Xenopus    IFFVSAKEVLNARIQKAQGMPEGGGALAEGFQARMFEFQNFERRFEECISQSAVKTKFEQHTKRAKHIAEVLRQIMESVHVAAQEQRCLCLEKCEEFKDR 400
Zebrafish  IFFVSAKEVLQARVQKAQGMPEAGGALAEGFQARMFEFQNFERRFEECISQSAVKTKFEQHTTRAKQISEALRLIMDSVHIAAQEQRIHCLEMKEEREDR 400
           **********.**:*********.*********.**********:******************* ***:*:*.:* **:*:*:**:**:   * *  ** ::*

    coiled coil domain
Cattle     LRFIDKQLELLAQDYKLRIKQITEEVERQVSTAMAEEIRRLSVLVDEYQMDFHPSPVVLKVYKNELHRHIEEGLGRNLSDRCSTAIASSLQTMQQDMIDG 500
Human      LRFIDKQLELLAQDYKLRIKQMTEEVERQVSTAMAEEIRRLSVLVDEYQMDFHPSPVVLKVYKNELHRHIEEGLGRNMSDRCSTAIASSLQTMQQDMIDG 500
Dog        LKFIDKQLELLAQDYKLRIKQITEEVERQVSTAMAEEIRRLSVLVDDYQMDFHPSPVVLKVYKNELHRHIEEGLGRNMSDRCSTAITNSLQTMQQDMIDG 500
Mouse      LRFIDKQLELLAQDYKLRIKQITEEVERQVSTAMAEEIRRLSVLVDDYQMDFHPSPVVLKVYKNELHRHIEEGLGRNMSDRCSTAISSSLQAMQQDMIDG 500
Rat        LRFIDKQLELLAQDYKLRIKQITEEVERQVSTAMAEEIRRLSVLVDEYQMDFHPSPVVLKVYKNELHRHIEEGLGRNMSDRCSTAITSSLQTMQQEMIDG 500
Xenopus    LEFIEKQLELLTEDYRFKIKLITDEVERQVSNAMAEEIRRLSVLVDEFQLDFHPSPVVLKVYKNELHRHIEEGLGRNLSDRCSSVISVSLQTTQQEMIEG 500
Zebrafish  LEFIDKQLDLLTLDCKSKIKKITEEVEKQVSNAMAEEIRRLAVLVDDFHMDFHPSPVVLKVYKNELHRHIEEGLGRNMSERCSSAITVALQTTQTDMIDG 500
           *.**:***:**: * : :** :*:***:***.*********:****::::***************************:*:***:.*: :*: * :**:*

Cattle     LKPLLPVSMRNQIDMLVPRQCFSLSYDLNCDKLCADFQEDIEFHFSLGWTMLVNRFLGPKNSRRALLGYSDQVQRPLPLTPANPSMPPLPQSSLTQEELM 600
Human      LKPLLPVSVRNQIDMLVPRQCFSLSYDLNCDKLCADFQEDIEFHFSLGWTMLVNRFLGPKNSRRALLGYNDQVQRPLPLTPANPSMPPLPQGSLTQEELM 600
Dog        LKPLLPVSVRSQIDMLVPRQCFSLNYDLNCDKLCADFQEDIEFHFSLGWTMLVNRFLGPKNSRRALMGYNDQVQRPIPLTPANPSMPPLPQGSLTQEEFM 600
Mouse      LKPLLPASVRSQIDMLVPRQCFSLSYDLNCDKLCADFQEDIEFHFSLGWTMLVNRFLGPKNSRRALMGYNDQVQRPIPLTPANPSMPPLPQGSLTQEELM 600
Rat        LKPLLPVSVRSQMDMLVPRQCFSLSYDLNCDKLCADFQEDIEFHFSLGWTMLVNRFLGPKNSRRALMGYSDQVQRPMPLTPANPSMPPLPQGSLTQEELM 600
Xenopus    MMPLLPPTARTQMDLLVPRQCFNLSYDLSCDKLCADFQEDVEFHFSLGWTMLVNRFLGPKNGRRALMAYNDQVPRVMPLTPVNPSMPPMPQGSMTQEELM 600
Zebrafish  LKPLLPITIREQVDKMVPRQCFALSYDLNCDKLCSDFQEDIGFHFSLGWTMLVNRFLGPKNTRRALMGYNDQVPRSLAITPVSTSMPPFPQGSMTQEELM 600
           : **** : * *:* :******* *.***.*****:*****: ******************* ****:.*.*** * :.:**...****:**.*:*****:*

    transmembrane domain   transmembrane domain
Cattle     VSMVTGLASLTSRTSMGILVVGGVVWKAVGWRLIALSFGLYGLLYVYERLTWTTKAKERAFKRQFVEYASEKLQLIISYTGSNCSHQVQQELSGTFAHLC 700
Human      VSMVTGLASLTSRTSMGILVVGGVVWKAVGWRLIALSFGLYGLLYVYERLTWTTRAKERAFKRQFVEYASEKLQLIISYTGSNCSHQVQQELSGTFAHLC 700
Dog        VSMVTGLASLTSRTSMGILVVGGVVWKAVGWRLIALSFGLYGLLYVYERLTWTTKAKERAFKRQFVEHASEKLQLVISYTGSNCSHQVQQELSGTFAHLC 700
Mouse      VSMVTGLASLTSRTSMGILVVGGVVWKAVGWRLIALSFGLYGLLYVYERLTWTTKAKERAFKRQFVEYASEKLQLIISYTGSNCSHQVQQELSGTFAHLC 700
Rat        VSMVTGLASLTSRTSMGILVVGGVVWKAVGWRLIALSLGLYGLLYVYERLTWTTKAKERAFKRQFVEYASEKLQLIISYTGSNCSHQVQQELSGTFAHLC 700
Xenopus    VSMVTGLASLTSRTSMGILVVGGVVWKAVGWRLIALSFGLYGLLYVYERLTWTNKAKERAFKRQFVDYAAEKLQLIVSYTGSNCSHQVQQELSSTFAHLC 700
Zebrafish  VSMVTGLASLTSRTSMGIIVVGGVIWKAVGWRLIALSVGLYGLLYVYERLTWTTRAKERTFKRQFVDYASEKLQLIVSYTGSNCSHQVQQELASTFSQLC 700
           ******************:******:************.*************** .:****:******::*:*****::***************:.**::**

coiled coil domain in bright green    22 amino acids lacking
HR2 domain in pale green              in aberrant cattle MFN2
Cattle     QQVDITRDNLEQEIAAMNKKVEALDSLQSRAKLLRNKAGWLDSELNMFTHQYLQPSR 757
Human      QQVDITRDNLEQEIAAMNKKVEALDSLQSKAKLLRNKAGWLDSELNMFIHQYLQPSR 757
Dog        QQVDVTRENLEQEIAAMNKKIEVLDSLQSKAKLLRNKAGWLDSELNMFTHQYLQPSR 757
Mouse      QQVDVTRENLEQEIAAMNKKIEVLDSLQSKAKLLRNKAGWLDSELNMFTHQYLQPSR 757
Rat        QQVDVTRENLEQEIAAMNKKIEVLDSLQSKAKLLRNKAGWLDSELNMFTHQYLQPSR 757
Xenopus    QQVDVTRENLEQDIDVLNKKIEILDSLQSKAKLLRNKAGWLDSELNMFTHQYLQQS- 756
Zebrafish  QQVDVTRQQLEDEIGDLNSKIEQLDTLQSKAKLLRNKAGWLDSELNMFIQQYLQQGR 757
           ****:**::**::*  :*.*:* **:***:****************** :**** .
```
